# Impact of mass distribution of insecticide-treated nets in Mozambique, 2012 to 2025: Estimates of child lives saved using the Lives Saved Tool

**DOI:** 10.1371/journal.pgph.0000248

**Published:** 2022-04-26

**Authors:** Erica A. Wetzler, Chulwoo Park, Jorge A. H. Arroz, Marta Chande, Figueiredo Mussambala, Baltazar Candrinho

**Affiliations:** 1 World Vision United States, Federal Way, Washington, United States of America; 2 Department of Public Health and Recreation, San José State University, San Jose, California, United States of America; 3 World Vision Mozambique, Maputo, Mozambique; 4 National Malaria Control Program, Maputo, Mozambique; Fundacao Oswaldo Cruz, BRAZIL

## Abstract

Malaria was the leading cause of post-neonatal deaths in Mozambique in 2017. The use of insecticide treated nets (ITNs) is recognized as one of the most effective ways to reduce malaria mortality in children. No previous analyses have estimated changes in mortality attributable to the scale-up of ITNs, accounting for provincial differences in mortality rates and coverage of health interventions. Based upon annual provincial ownership coverage of ITNs, the Lives Saved Tool (LiST), a multi-cause mathematical model, estimated under-5 lives saved attributable to increased household ITN coverage in 10 provinces of Mozambique between 2012 and 2018, and projected lives saved from 2019 to 2025 if 2018 coverage levels are sustained. An estimated 14,040 under-5 child deaths were averted between 2012 and 2018. If 2018 coverage levels are maintained until 2025, an additional 33,277 child deaths could be avoided. If coverage reaches at least 85% in all ten provinces by 2022, then a projected 36,063 child lives can be saved. From 2012 to 2018, the estimated number of lives saved was highest in Zambezia and Tete provinces. Increases in ITN coverage can save a substantial number of child lives in Mozambique. Without continued investment, thousands of avoidable child deaths will occur.

## Introduction

**Fig 1:**
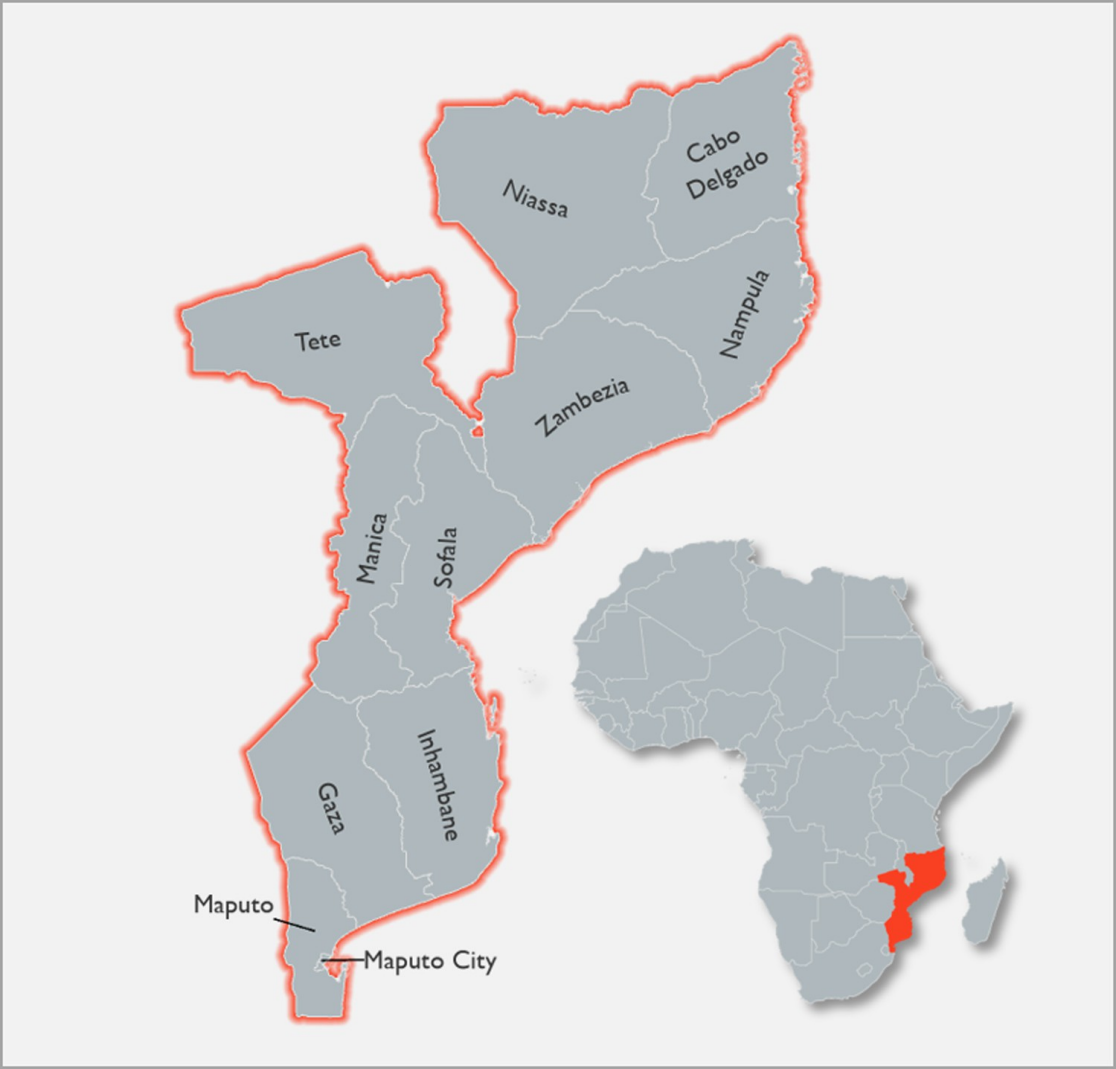
Provincial map of Mozambique [13, 14]. Source: **Africa Continental Base map:** https://www.naturalearthdata.com/downloads/10m-cultural-vectors/10m-admin-0-details/; **Subnational Boundaries:** https://data.humdata.org/dataset/mozambique-administrative-levels-0-3.

According to the World Health Organization (WHO)’s World Malaria Report 2021, Mozambique has the fourth highest burden of malaria worldwide, with an estimated 10 million cases in 2020 [1]. There were an estimated 49,913 post neonatal deaths in 2017 in Mozambique, with 10,132 deaths due to malaria, making it the top cause of post neonatal deaths [2]. Malaria is endemic throughout the country, and malaria prevalence has historically been greater in the northern provinces of the country, where there are more humid climates and higher populations (**Fig 1**) [3, 4].

The most important malaria vectors in Mozambique are *Anopheles funestus*, *A*. *gambiae* sensu stricto (*s*.*s*.) and *A*. *arabiensis* [5–8]. *A*. *merus* has been reported as playing secondary role on malaria transmission in the coastal regions [9]. Biting times and preference for indoor or outdoor biting varies across species and regions, and has changed in response to prolonged use of insecticide treated nets (ITNs) [10, 11]. Recent research in neighboring Malawi shows that *Anopheles gambiae* s.l. biting activity was highest in the late evening hours during the wet season and was more likely to bite outdoors [12]. *Anopheles funestus* biting activity was highest during late evening in the dry season, and early morning in the wet season.

Mozambique’s National Strategic Plan for Malaria (2017–2022) focuses on reducing the malaria burden in high endemic areas while sustaining gains in low transmission areas to accelerate elimination. The plan emphasizes strengthening case management, universal access to diagnosis and treatment, strengthening malaria surveillance systems, and the continuation of universal coverage of long last insecticidal nets (LLINs) as the main vector control strategy, with the use of indoor residual spraying (IRS) as a mitigation method in areas where there is documented pyrethroid resistance [15].

There is substantial evidence linking use of LLINs and their precursors, ITNs, with reduced child mortality and morbidity [16]. Universal coverage of LLINs is a core malaria prevention strategy, and part of the first pillar of WHO’s Global Technical Strategy for Malaria 2016–2030 [17]. Globally, LLINs have become a core intervention of national control strategies with over 2.3 billion nets distributed worldwide from 2009 through the end of 2020 [18].

The National Malaria Control Program’s (NMCP) current objectives relevant to ITN distribution are to ensure that at least 85% of registered households own an ITN. The NMCP’s goal is to distribute one ITN for every 1.8 people in the household every three years [15]. Through funding from the U.S. government’s President’s Malaria Initiative, and later from the Global Fund for Malaria Tuberculosis and AIDS, the NMCP worked with partners to organize mass distributions of ITNs, starting within selected districts in some provinces. Between 2011 and 2020, over 48 million bed nets were distributed nationwide, including the first countrywide campaign in 2017 that delivered over 16 million ITNs to 95% of registered households [19]. The 2020 campaign planned to deliver over 12 million ITNs to seven provinces but was initially delayed by the COVID-19 pandemic. However, door-to-door distribution took place during the latter half of the year. The NMCP also estimates that about 1.8 million ITNs are distributed each year to pregnant women during antenatal consultations [15].

Ownership and use of ITNs and LLINs in Mozambique increased between 2011 and 2018; the percentage of households that reported owning at least one ITN was 51.4% in 2011 and 82.2% in 2018. Those households reporting owning at least one ITN for every two people in the household and sleeping under the ITN the night before the survey increased from 22.6% in 2011 to 51.2% in 2018 [20, 21]. the gap between household ownership and use of ITNs varied by province, and by 2018, was greatest in Niassa province in the north (84.1% ownership compared to 32.9% use) and least in Maputo province in the south (80.4% ownership compared to 62.6% use) [20]. In 2011, the NMCP developed a national strategy to expand distribution of ITNs.

Typically, the impact of ITNs is measured through outcomes such as household ownership and access, which estimates the proportion of the population potentially covered by existing ITNs, assuming that each ITN in a household can be used by two people [22]. There has been less focus on the impact of ITNs related to mortality or morbidity, and most countries where malaria is endemic do not have reliable data on malaria mortality trends [23].

The Lives Saved Tool (LiST), developed by Johns Hopkins University, is a modeling application available through the Spectrum software package that estimates child mortality and “lives saved” for children under 5 years of age using the age structure of the target population, fertility rates, under-5 mortality rates, cause of death structure, and changing coverage estimates of key child survival and maternal interventions [24]. LiST is characterized by a linear, deterministic mathematical model that describes relationships between fixed inputs, namely intervention coverage and outputs, including “lives saved,” and child and maternal mortality [23–25]. LiST has been used widely to model the impact of changing coverage of key maternal and child health interventions on child and maternal mortality [26].

Several examples comparing estimates from LiST to measured changes in child mortality from field studies include: comparing child mortality directly measured through a maternal and child health project in Mozambique with LiST estimates [27], which found that LiST gave reasonably accurate estimates of the infant and child mortality decline in an area where a package of community-based interventions was implemented. Another analysis that compared reductions in all-cause child mortality estimated by LiST to four studies that estimated changes in in all-cause mortality following scale-up of vector control interventions found that the LiST model did not systemically under- or over-estimate the impact of ITNs on all-cause child mortality; LiST also performed reasonably well at estimating the effect of vector control scale-up [28]. Further, other studies showed that the LiST methodology could be reasonably applied in various countries for vector control interventions, supporting use of LiST as an aid for program planning to tailor packages of community-based interventions to the epidemiological context [28–30].

By 2012, Mozambique was distributing nearly all LLINs (rather than ITNs); however, to maintain consistency and because the main unit of analysis in LiST is ITNs, we refer to ITNs in this paper. A number of previous studies have used LiST to analyze changes in coverage of LLINs even though the unit of analysis in LiST is ITNs [29, 31, 32]. In Mozambique, previous analyses using LiST have modeled the direct effect of the scale-up of malaria interventions (IRS, household ITN ownership, Intermittent Prophylactic Treatment (IPTp) during pregnancy, and treatment of uncomplicated malaria) on reducing malaria-specific mortality from 2007 to 2011, and between 1997 and 2011 [32, 33].

However, there are no known studies or meta-analysis that have estimated changes in child mortality attributable to the distribution and scale-up of household ITN or LLIN ownership in Mozambique, accounting for heterogenous mortality rates, differences in disease patterns, and varying coverage of maternal and child health interventions between provinces. Modeling mortality outcomes, including lives saved, can help stakeholders to allocate limited resources more strategically and advocate for continued investment in ITNs as a cornerstone for driving and maintaining reductions in under-5 child mortality in Mozambique [34].

This study aimed to estimate deaths averted in children aged under-5 due to ITN distributions in 10 provinces of Mozambique between 2012 and 2018, and project the number of expected lives saved in children aged under-5 from 2019 to 2025 if 2018 provincial household ITN coverage levels remain constant or are increased to at least 85% by 2022.

## Materials and methods

The LiST module (Version 5.761) was used to estimate the impact of ITNs on under-5 lives saved, using provincial changes in household ITN ownership from national household surveys in 2011, 2015, and 2018 [35–37]. LiST is computer-based software, using a deterministic mathematical model to describe relationships between inputs (coverage of interventions) and outputs (cause-specific mortality, such as neonatal or children 1–59 months, or “lives saved”). Under the basic model structure, the proportional reduction in mortality for a specific child cause of death by a specific intervention is a function of: 1) the effectiveness of the intervention, 2) the increase in coverage of the intervention and 3) the affected fraction, adjusted for the unrealized potential impact [38]. The modeling equations behind the basic LiST models for estimations of lives saved and the herd effect of bed nets are detailed in Winfrey et al. [38]. The primary assumption in LiST is that mortality rates and cause of death structures do not change dynamically, and that differences will be in response to changes in intervention coverage [39].

LiST runs through Spectrum, a suite of integrated modules maintained by Avenir Health with guidance from Johns Hopkins Bloomberg School of Public Health [40]. To produce projections, LiST incorporates detailed demographic information through linkages with demographic and family planning modules, also housed in Spectrum; country-specific cause of death information for children under-5 and mothers; health and nutrition status; coverage of key health interventions; and effectiveness of interventions from scientific review. For background population information, LiST utilizes the most recent population projections from the United Nations Population Division [41], demographic and intervention coverage data from Demographic and Health Survey (DHS), and Multiple Indicator Cluster Survey (MICS), which are automatically loaded into the LiST module in Spectrum. There are over 70 separate interventions in LiST [24], and evidence for efficacy and effectiveness of interventions used in the model is guided by the Child Health Epidemiology Reference Group (CHERG), which has developed rules of evidence to decide what interventions should be in the model and how to develop estimates of efficacy and effectiveness that are used in the model [42].

For this study, the intervention indicator of interest in LiST was defined as households owning at least one ITN or protected by IRS [43]. In a context such as Mozambique where data on ITN use and ownership are only available through sporadic household surveys, ITN possession rather than ITN use is the preferred indicator because: 1) household possession of ITNs captures the community effect better than individual use; 2) ITN use measured through DHS and UNICEF MICS surveys are implemented in the dry season, which may underestimate effects during higher transmission wet seasons; 3) use last night is likely the lowest measure of regular use over time; and 4) use by children under age 5 years likely underestimates use by anyone in the household [44].

Separate LiST models for each province in Mozambique were needed due to provincial differences in cause of death structures and varying levels of availability and use of the maternal and child health interventions projected by LiST. Standard sub-national (provincial) profiles from the LiST website had the default initial parameter values for the interventions in the LiST model. When possible, these were updated with values from the 2011 Mozambique DHS for baseline coverage of some interventions. Provincial neonatal and under-5 and infant mortality rates were entered manually for each province in the baseline year, 2011, from the 2011 Mozambique DHS. Malaria mortality rates for 2011 for each province were derived by applying the proportion of post-neonatal deaths under-5 years due to malaria (among other causes) [45]. Effectiveness of ITNs and/or IRS, defined as the percent of deaths due to a specific cause that are reduced by ITNs or IRS, was estimated and included in the LiST models as children 1–59 months living in households protected by ITNs and/or IRS having a 55% lower risk of malaria-attributable death, or 55% protective efficacy [44].

DHS data were used for baseline ITN coverage in each province in 2011 [36]. To estimate trends in percentage of households owning ITNs between 2011 and 2018, we entered the provincial ITN household ownership coverage for 2015 from the AIDS Indicator Survey and the 2018 Malaria Indicator Survey, and then interpolated linearly between years [32, 46]. Number of children’s lives saved per year due to increases in percentage of households owning ITNs and their 95% confidence intervals were estimated based on the annual ITN coverage from household surveys compared to 2011 coverage levels. Coverage of all other (non-ITN) interventions was kept constant throughout the time periods considered in the models.

LiST uses deterministic sensitivity analysis to assess how model results are sensitive to parameter values. When LiST produces model outputs, this process repeats three times, each with the estimate of effectiveness of all interventions, the lower confidence interval, and the higher confidence interval. The sensitivity analysis produces the lower bound, estimate, and upper bound outputs [47]. For this study, the sensitivity analysis used 95% confidence intervals around the effectiveness of the primary intervention of interest, household ITN ownership, to estimate the range around the model output, the estimated number of child lives saved) [47]. For ITN effectiveness, the lower bound was set at 49%; the upper bound was 61%; and the estimate was 55% [44].

Between 2019 and 2025, we estimated lives saved based on two ITN household ownership scenarios: 1) 2018 coverage levels were held constant, and 2) the NMCP’s most recent strategy of increasing household ownership to 85% by 2022. In the second scenario, for provinces below 85% coverage in 2018, coverage was set to 85% in 2022, and then linearly interpolated between 2018 and 2022. From 2022 to 2025, 85% ownership coverage was kept constant in these provinces. For the five provinces with over 85% coverage in 2018, their 2018 coverage levels were kept static from 2019 to 2025.

The study population was 22 million people in Mozambique in 2011, including 17.45% of the population under 5 years of age, or about 3.84 million children [48]. The 2011 census data were used as the base year, and LiST adjusted the population size over time from 2011, reflecting population growth rates. Maputo City, the urban area that lies south of Maputo province, was excluded for the following reasons: 1) in Maputo City, ITN distribution was not the primary malaria prevention intervention and 2) ITNs have not been distributed there by the NMCP since 2017.

This research uses secondary data and is not subject to ethics approval.

## Results

### Provincial level changes in household ITN coverage in Mozambique

In 2011, the percentage of households owning an ITN was lowest in Maputo province (37.6%) in the south, and highest in Cabo Delgado province (61.3%) in the north (**Table 1**). By 2015, coverage had increased in all ten provinces, except Manica, which decreased from 53.9% to 47.8%. Household ownership coverage was nearly flat in Nampula province between 2011 and 2015, increasing from 60.5% to 61.0%. By 2018, all provinces except Sofala were over 80% ownership coverage, with Inhambane and Gaza in the south over 90% and Cabo Delgado in the northernmost part of the country at 94.3%. From 2011 to 2018, Gaza and Maputo provinces had the largest percentage point (pp) increase in household ownership, 46.2 pp and 42.8 pp respectively. Sofala province only increased by 10.3 pp in the same time period. These trends, along with provincial populations and cause of death structures, provided the foundation for examining estimated lives saved in each province over time.

**Table 1:** Trends in household ownership coverage of ITNs in 10 provinces of Mozambique in 2011, 2015, and 2018, from the 2011 DHS, 2015 AIS, and 2018 MIS.

Province	Coverage 2011 n (%)	Coverage 2015 n (%)	Coverage 2018 n (%)	Percentage point change in coverage, 2011–2018
Gaza	333 (46.0)	424 (71.4)	310 (92.2)	**46.2**
Maputo Province	355 (37.6)	280 (71.0)	298 (80.4)	**42.8**
Tete	774 (47.3)	411 (70.3)	496 (87.2)	**39.9**
Inhambane	471 (53.9)	417 (86.3)	367 (91.4)	**37.5**
Niassa	392 (47.2)	248 (67.0)	289 (84.1)	**36.9**
Zambezia	1,167 (46.5)	510 (56.8)	992 (80.7)	**34.2**
Manica	494 (53.9)	262 (47.8)	322 (87.4)	**33.5**
Cabo Delgado	721 (61.3)	581 (77.2)	505 (94.3)	**33**
Nampula	1,549 (60.5)	981 (61.0)	1032 (80.8)	**20.3**
Sofala	630 (56.8)	371 (62.0)	313 (67.1)	**10.3**

### Estimated lives saved in children under-5 from 2012 to 2018

**Fig 2:**
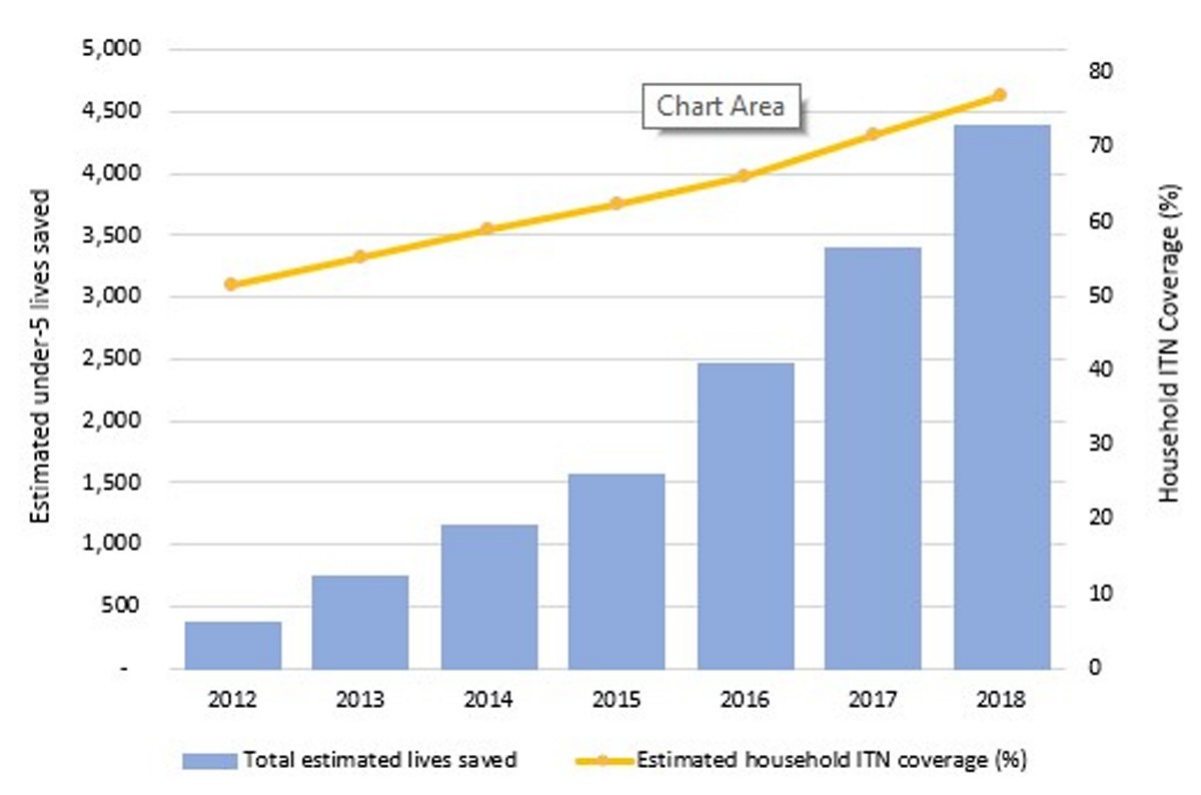
Mean household ownership coverage of ITNs and total estimated lives saved of children under-5 due to ITN ownership in 10 provinces of Mozambique, 2012 to 2018.

**Fig 3:**
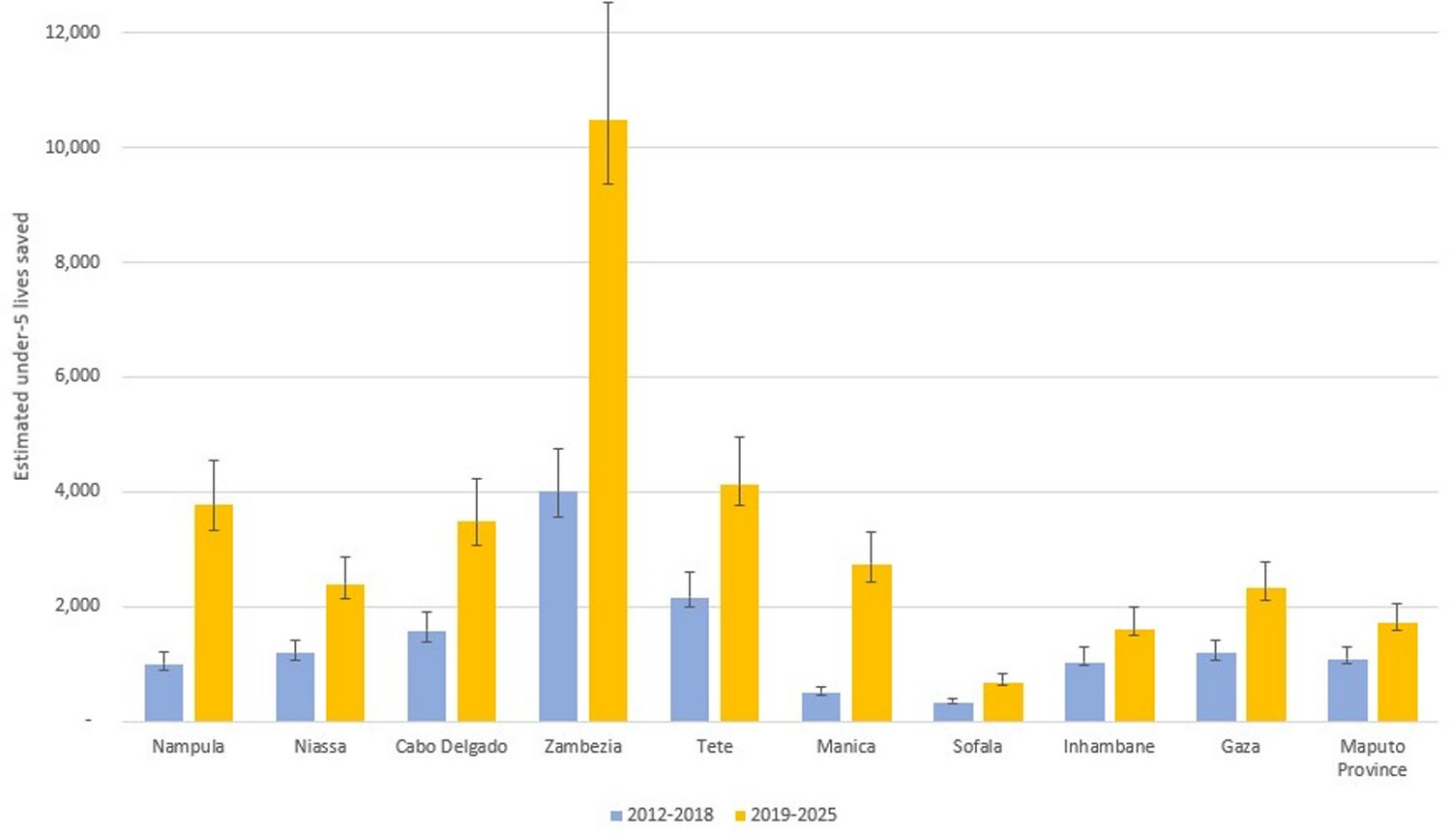
Estimated number of under-5 lives saved by province in Mozambique from 2012 to 2018 based on changes in household ITN ownership coverage.

In total, we estimate that 14,040 lives of children under-5 were saved due to household ownership of ITNs between 2012 and 2018. The estimated number of under-5 lives saved nationwide increased every year starting from 368 in 2012 to 1,554 in 2015 and reached 4,379 in 2018 (**Fig 2**). Provincial level comparisons from 2012 to 2018 shown in **Fig 3** reveal that Zambezia (3,997), followed by Tete (2,163) province had the most lives saved attributable to ITN distribution. From 2012 to 2018, the estimated under-5 deaths averted in Zambezia province alone were about 28.5% of the country total for the same time period. Sofala province had the fewest estimated child lives saved at 323 between 2012 and 2018. In Manica province, we estimated that child lives were lost rather than saved between 2012 and 2015, owing to the reduction in ITN coverage in the province during those years from 53.9% in 2011 to 47.8% in 2015.

### Projected under-5 lives saved from 2019 to 2025

**Fig 4:**
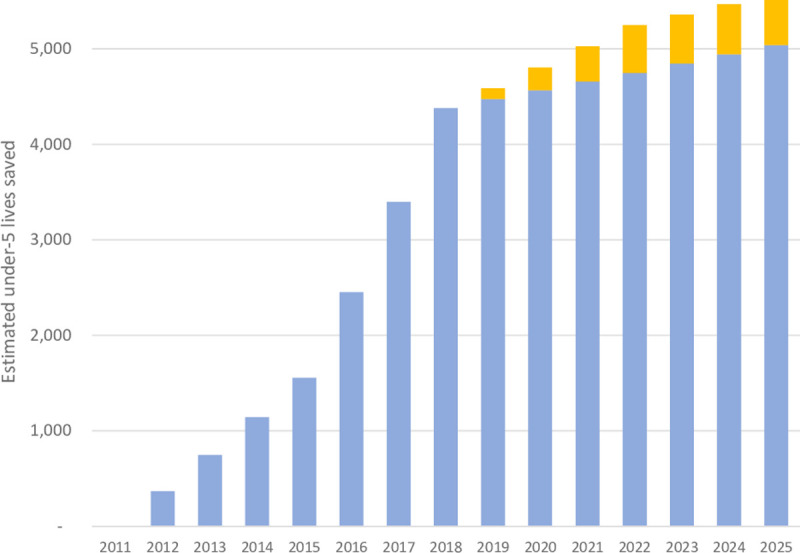
Estimated additional under-5 lives saved based on changes in household ownership coverage of ITNs, 2012–2025. The blue bars show maintaining 2018 coverage levels through 2025. The orange bars show the projected number of under-5 lives saved if provinces with ≤85% household ITN coverage reach 85% by 2022 and maintain 85% coverage until 2025, and if the 5 provinces that exceeded 85% coverage by 2018 maintain their 2018 coverage levels through 2025.

If 2018 household ownership coverage levels remain constant, an estimated 33,277 additional child deaths will be avoided from 2019 to 2025 in all 10 provinces, with 5,038 deaths averted in 2025 alone (**Fig 4**). Five provinces reached at least 85% ownership coverage by 2018 (Cabo Delgado, Tete, Manica, Inhambane, and Gaza). From 2019 to 2025, if 2018 coverage levels remain constant, an additional 10,480 lives are projected to be saved in Zambezia province alone, accounting for 31.5% of the total estimated lives saved, followed by Tete (4,113) and Nampula (3,771) provinces, with the fewest lives saved in Sofala.

If all ten provinces reach at least 85% coverage by 2022 and maintain 85% coverage until 2025, an additional 2,786 under-5 lives are expected to be saved, besides the 33,277 already expected with 2018 coverage levels, for a total of 36,063 estimated deaths avoided between 2019 to 2025 due to household ITN distribution (**Fig 4**). Complete data tables of estimated under-5 lives saved by year and by province can be found as S1 and S2 Tables.

#### Sensitivity analysis

For each province, we used LiST to approximate the 95% confidence intervals for the upper and lower bounds on the point estimate of the total number of deaths averted of children under-5 from 2012 to 2018, and 2019 to 2025 (**Table 2**). From 2012 to 2018, the percent difference between the lower bound and the point estimate was about 10% (range by province: 7.78%-12.40%), while the difference between the point estimate and the upper bound was about 20% (range by province: 18.98%-24.54%). Provincial ranges were similar for the period between 2019 to 2025.

**Table 2:** Sensitivity analysis with 95% confidence intervals around the effectiveness of ITN/IRS coverage in order to estimate the upper and lower bounds on the number of child lives saved, by province, 2012–2018 and 2019–2025.

	2012–2018					2019–2025				
Province	Estimated under-5 lives saved	Lower bound	% Difference	Upper bound	% Difference	Estimated under-5 lives saved	Lower bound	% Difference	Upper bound	% Difference
Nampula	1,001	885	11.59	1,209	20.78	3,771	3,331	11.67	4,558	20.87
Niassa	1,185	1,061	10.46	1,410	18.99	2,395	2,148	10.31	2,860	19.42
Cabo Delgado	1,573	1,378	12.40	1,901	20.85	3,484	3,054	12.34	4,219	21.10
Zambezia	3,997	3,570	10.68	4,763	19.16	10,480	9,362	10.67	12,522	19.48
Tete	2,163	1,983	8.32	2,600	20.20	4,113	3,771	8.32	4,961	20.62
Manica	506	444	12.25	604	19.37	2,745	2,412	12.13	3,289	19.82
Sofala	323	295	8.67	393	21.67	677	616	9.01	822	21.42
Inhambane	1,031	966	6.30	1,284	24.54	1,593	1,489	6.53	1,987	24.73
Gaza	1,181	1,075	8.98	1,417	19.98	2,310	2,097	9.22	2,775	20.13
Maputo Province	1,080	996	7.78	1,285	18.98	1,709	1,577	7.72	2,037	19.19
**TOTAL**	14,040	12,653	9.88	16,866	20.13	33,277	29,857	10.28	40,030	20.29

## Discussion

The scale up of ITNs in Mozambique is estimated to have saved an additional 14,040 lives of children under-5 from 2012 to 2018, with an expected additional 33,277 lives saved between 2019 and 2025 if household ITN ownership coverage levels remain constant at 2018 levels. The increase in coverage of ITNs between 2011 and 2018 was greatest in Maputo province. However, the greatest estimated number of child deaths avoided in this time period were in Zambezia and Tete provinces, likely because these provinces had relatively larger populations of children under-5 compared to other provinces and relatively greater increases in the percentage of households owning ITNs from 2011 to 2018, 34.2 pp and 39.9 pp increase respectively. Zambezia is the second most populated province in Mozambique at 5,002,457 [49]. Though Nampula province has the largest provincial population in the country, the increase in ITN coverage between 2011 and 2018 was second lowest among the 10 provinces at 20.3 pp, explaining why there are more projected lives saved in Zambezia. From 2011 to 2018, there was a consistent increase in ITNs distributed in the country, culminating in 95% ITN coverage of registered households by the end of 2017, which had not been previously been accomplished [19]. If coverage levels are maintained, more children’s lives can be saved, especially as the population grows.

Notably, the distance between the estimate and the upper bound of the 95% confidence intervals for the number of child lives saved was usually about double that of the distance between the lower bound and the estimate. Since the trials used to estimate effectiveness were likely biased toward the null due to the beneficial effects bestowed upon unprotected children in control villages as a result of the community effect, LiST underestimates the true impact of ITN effectiveness on child mortality, explaining in part why the upper bound for the number of deaths averted is consistently farther from the point estimate than the lower bound [44].

This research adds to the body of evidence showing the relative importance of ITNs to prevent under-5 deaths in Mozambique. Though other studies have used slightly different approaches and did not assess the effects of ITNs only or investigate provincial differences in lives saved, their findings also demonstrate the importance of ITNs in preventing deaths of children in Mozambique [31–33]. The President’s Malaria Initiative conducted an analysis estimating deaths prevented due to the scale-up of malaria control interventions from 2007 to 2011 and estimated that 13,198 (range: 9,231 to 17,231) deaths were prevented in children aged 1 to 59 months due to the scale-up of ITNs and IRS [33]. In an analysis of 11 interventions, Macicame et al. estimated that 55,757 under-5 child lives were saved between 1997 and 2011 (15 years) through ITNs and IRS. Compared to ITN ownership, only reductions in child wasting prevalence saved more lives [32].

Junior et al. used historical data on coverage of maternal and child health interventions together with mathematical modeling to project future coverage levels for 22 health interventions from 2015 to 2030, and then used LiST to estimate additional child lives saved and changes in the national child mortality rate over time [31]. They concluded that the two interventions projected to save the most under-5 lives between 2015 and 2030 were increased coverage of artemisinin-based combination therapies (ACTs) and ITN ownership, contributing to an estimated 40.9% of the total under-5 lives saved if historical trends continue [31]. This study considered changes in coverage of other maternal and child health interventions.

In countries such as Mozambique where impact indicators specific to distribution of ITNs are not routinely assessed, NGOs, government partners, researchers, project planners have used lives saved estimates from LiST for advocacy, evaluation, and strategic planning. As such, this study can help to inform decision-making around malaria control policies and planning programs in Mozambique, suggesting changes or continuations to policies or contributing to the development of stronger malaria prevention programs in the country by identifying the highest-impact interventions in a given epidemiological setting [50].

Several strengths of LiST are that it has been validated by its authors against observed field data [28, 51, 52] and it incorporates a comprehensive approach to mortality modeling, allowing calculation of estimated lives saved from increases in ITNs while coverage levels of other interventions remain static. However, this study had some limitations. First, LiST uses a linear deterministic model, but many infectious diseases such as malaria are characterized by non-linear transmission dynamics. LiST cannot reflect non-linear transmission, but it is not intended to model disease transmission. Rather, it models lives saved based on changes in coverage, intervention effectiveness and effected fractions. Therefore, LiST’s linear projection is an appropriate alternate method and has the advantage of incorporating coverage for multiple maternal and child health interventions into a single model. Given the dearth of routine ITN coverage data and cause-specific mortality data, LiST is an adequate substitute when real world mortality data are unavailable.

Coverage of IRS was not included in this analysis. In 2019, the IRS program covered five provinces, or about 5.7 million people, which would have contributed to further gains in lives saved. However, all provinces covered by IRS were also covered by ITNs [53]. Furthermore, other malaria interventions such as case management with artemisinin-based combination therapy (ACTs) and chemoprophylaxis for pregnant women were considered static for this analysis. Since the LiST tool focuses on children under-5 years of age and women of reproductive age, this analysis focused only on children under-5, underestimating the full impact of ITN distribution on the population of Mozambique. However, children under-5 carry the greatest overall and malaria mortality burden. The protective efficacy of ITNs used in the model does not consider increasing pyrethroid resistance after 2010, which has been documented in multiple regions of the country [54–56]. Taking into account pyrethroid resistance documented in the last ten years would shift the protective efficacy downward, resulting in fewer lives saved projected by the models.

Multiple analyses have shown that continued interventions to prevent childhood malaria, especially ITNs, can result in substantial reductions in child mortality and save lives. The total estimated cost per ITN in Mozambique was $1.22 in 2017 (including procurement, logistics, training, transport), a relatively small investment given the number of lives that can be saved [19]. Further, sustaining or increasing coverage of ITNs may be more feasible than scaling other key child health interventions such as malaria treatment with ACTs and oral rehydration solution for diarrhea. In an analysis of 69 countries, Walker et al. predicted that by 2035, the coverage of four interventions all related to malaria prevention, including household ownership of ITNs, will increase rapidly, while another 24 interventions will not see substantial scale-up, bolstering the case for continued investment in mass distribution of ITNs [24].

Gaps in coverage of maternal and child health interventions, including ITN distribution, need to be viewed through an equity lens, which acknowledges the need for a multi-intervention approach, while strategically focusing on those interventions known to be effective at reducing mortality, and whose distribution and delivery mechanisms promote equity (lower cost, less time for distribution and training). Mass ITN campaigns are last-mile solutions to help close the equity gap. In Mozambique, they can be distributed on an ongoing basis, usually through ante-natal consultations, but are primarily distributed through mass campaigns every few years, which reduces costs, and brings them to the most remote areas, often the most vulnerable. In contrast to other maternal and child health interventions, ITNs benefit the entire household. Logistical burdens surrounding transport and handling are also less complex, depending on fewer intermediaries. Mass distributions are one-off events rather than continuous, reducing the number of trips needed for delivery. Over time, this makes mass distribution events less vulnerable to the effects of external factors such as emergencies and political instability that have undermined sustaining or increasing coverage of other maternal and child health interventions. However, there are challenges associated with distribution, access, and use of ITNs at the household level that should be considered during allocation of scarce financial resources for child health. This analysis shows that child deaths are concentrated in provinces in the north and central regions. If funding gaps continue, targeted selection of high burden, more populated provinces and districts should be prioritized.

This study estimated 14,040 under-5 lives in Mozambique were saved due to household ITN ownership from 2012 to 2018, and 33,277 child deaths can be averted between 2019 and 2025 if ITN ownership coverage levels remain constant at 2018 levels. Increasing household ownership coverage of ITNs is clearly a strategic opportunity for saving child lives but will require substantial resource mobilization. Without continued investment, thousands of avoidable child deaths will occur.

## Supporting information

S1 Table Estimated under-5 lives saved by province from 2012 to 2025. The estimated child lives saved are based on changes in household ITN coverage from 2012 to 2018, and two scenarios for maintaining or increasing coverage from 2019 to 2025. Scenario 1 sustains 2018 ITN coverage levels from 2019 to 2025, while Scenario 2 increases 2018 ITN coverage to 85% by 2022 for provinces not yet at 85% in 2018; for provinces with coverage greater than 85% in 2018, coverage is maintained at 2018 levels until 2025.
(XLSX)

S2 Table Lives Saved Tool (LiST) output for estimated deaths averted of children under-5 years old, by province and year from 2011 to 2025. Estimates for additional child lives saved attributable to increased ownership of ITNs at household level from 2011 to 2018 compared to maintaining 2011 ITN ownership coverage, with upper and lower bounds for 95% confidence intervals, and disaggregated by <1 month of age and 1–59 months.
(XLSX)
